# The inhibition of the Human Immunodeficiency Virus type 1 activity by crude and purified human pregnancy plug mucus and mucins in an inhibition assay

**DOI:** 10.1186/1743-422X-5-59

**Published:** 2008-05-19

**Authors:** Habtom H Habte, Corena de Beer, Zoë E Lotz, Marilyn G Tyler, Leann Schoeman, Delawir Kahn, Anwar S Mall

**Affiliations:** 1 Department of Surgery, University of Cape Town, Cape Town, South Africa; 2 Discipline of Medical Virology, University of Stellenbosch and National Health Laboratory Service, Tygerberg Business Unit, Stellenbosch, South Africa; 3 Obstetrics and Gynaecology, University of Cape Town, Cape Town, South Africa

## Abstract

**Background:**

The female reproductive tract is amongst the main routes for Human Immunodeficiency Virus (HIV) transmission. Cervical mucus however is known to protect the female reproductive tract from bacterial invasion and fluid loss and regulates and facilitates sperm transport to the upper reproductive tract. The purpose of this study was to purify and characterize pregnancy plug mucins and determine their anti-HIV-1 activity in an HIV inhibition assay.

**Methods:**

Pregnancy plug mucins were purified by caesium chloride density-gradient ultra-centrifugation and characterized by Western blotting analysis. The anti-HIV-1 activities of the crude pregnancy plug mucus and purified pregnancy plug mucins was determined by incubating them with HIV-1 prior to infection of the human T lymphoblastoid cell line (CEM SS cells).

**Results:**

The pregnancy plug mucus had MUC1, MUC2, MUC5AC and MUC5B. The HIV inhibition assay revealed that while the purified pregnancy plug mucins inhibit HIV-1 activity by approximately 97.5%, the crude pregnancy plug mucus failed to inhibit HIV-1 activity.

**Conclusion:**

Although it is not clear why the crude sample did not inhibit HIV-1 activity, it may be that the amount of mucins in the crude pregnancy plug mucus (which contains water, mucins, lipids, nucleic acids, lactoferrin, lysozyme, immunoglobulins and ions), is insufficient to cause viral inhibition or aggregation.

## Background

Cervical mucus is reported to regulate sperm penetration and transport to the upper reproductive tract [1,2]. It also provides lubrication to the cervix by enhancing its wetness and thus preventing its desiccation, and retards enzymatic degradation of the cervix and providing it with protection from pathogenic invasion and infection [3-5]. Its secretion, at a rate of 20–60 mg per day acts as a fence to sperm and pathogen entrance [6]. Although a reduction in mucus viscosity may allow foreign agent penetration, millions of micro-organisms a day are reported to be cleared from the reproductive tract by cervical secretions that are the tract's most effective first line of defence [7].

Thus far six mucin genes have been reported to be expressed by the female reproductive tract, namely MUC1, MUC2, MUC4, MUC5AC, MUC5B and MUC6 [6]. The genes for MUC2, MUC5B, MUC5AC and MUC6, are found on chromosome 11p15.5 and express the secreted gel forming mucins, whereas MUC1 and MUC4 are membrane associated mucins expressed by the epithelium of the ecto-cervix and vagina [7]. Of these, MUC4 and MUC5B are reported to be the major mucin genes expressed by the endo-cervix [8]. The variation, under hormonal influence, of the viscoelastic and rheological properties of these mucins during the menstrual cycle is well documented [4].

Human crude saliva is known to inhibit Human Immunodeficiency Virus type 1 (HIV-1) activity in an *in vitro *assay [9,10]. These authors speculated that it was the mucus component that inhibited the virus. We very recently showed that both crude saliva and its purified mucin components MUC5B and MUC7 inhibited HIV-1 activity [11] and so did the purified MUC1 of breast milk [12]. The MUC1 of breast milk also showed anti-pox viral activity [13]. Our hypothesis is that cervical mucins should have a similarly inhibitory effect on HIV-1 activity, an important question considering that the vagina and cervix are significant routes for HIV transmission. The aim of this study therefore was to extract and purify the mucins in the pregnancy plug mucus and to determine their anti-HIV-1 activity using an HIV inhibition assay.

We therefore extracted and purified mucins from the pregnancy plug mucus which occludes the cervical canal throughout the pregnancy period [2,14]. This large mucus plug which is more like the mucus of the luteal phase than the mucus of the mid-cycle [2] was obtained during labour and just prior to delivery.

Sub-Saharan Africa is reported to be home to about 25 million adults and children who are HIV positive [15]. In Southern Africa 25.7% of the population has HIV/AIDS, making this the most highly prevalent region of infection compared to the Eastern and the Western regions with 11.4% and 4.3% prevalence respectively [16]. In South Africa alone, between 4.68 and 7.03 million people were living with HIV/AIDS in 2004 [17], of whom 55% were female [18]. Thus this preliminary study could make a significant contribution to the efforts being made in controlling this epidemic.

In this study we report the anti-HIV-1 activities of crude and purified human pregnancy plug mucus and mucins in an *in vitro *inhibition assay. We have demonstrated that the purified mucins from the pregnancy plug mucus inhibited HIV-1 infection of the CEM SS cells. However, the crude pregnancy plug mucus failed to inhibit HIV-1 infection of these cells.

## Results

### Mucin purification

**Figure 1:**
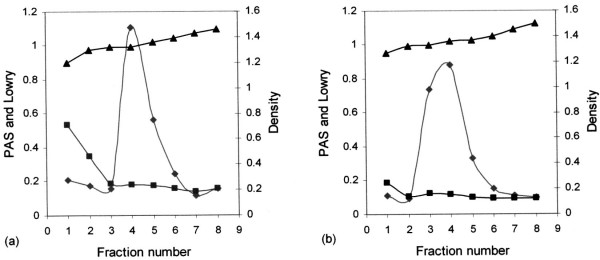
**Caesium chloride density gradient purification of the pregnancy plug mucins.** Samples in 4 M GuHCl were adjusted to a density of 1.39 to 1.40 g ml^-1 ^with solid caesium chloride. Density gradient centrifugation was performed in a Beckman L45 ultra-centrifuge for 48 h at a 105 000 g at 4°C. Mucin positive fractions (◆) at a density (▲) between 1.37–1.42 and still associated with some protein (■) (a) were pooled and prepared for the second step centrifugation (b). Finally fractions (fraction number 3, 4 and 5) were pooled, dialysed against three changes of distilled water and freeze-dried.

Pregnancy plug mucins were purified by density gradient centrifugation, twice in caesium chloride/4 M GuHCl with a buoyant density between 1.39 and 1.40 g/ml to remove proteins and nucleic acids. The purification profile in Fig. 1 demonstrates a clear separation of the lower density proteins positive for Lowry from the higher-density glycoproteins positive for PAS. The mucin-rich fractions (fractions number 3, 4 and 5) (Fig. 1b) were pooled, dialysed against three changes of distilled water and freeze-dried.

### SDS-PAGE analysis

**Figure 2:**
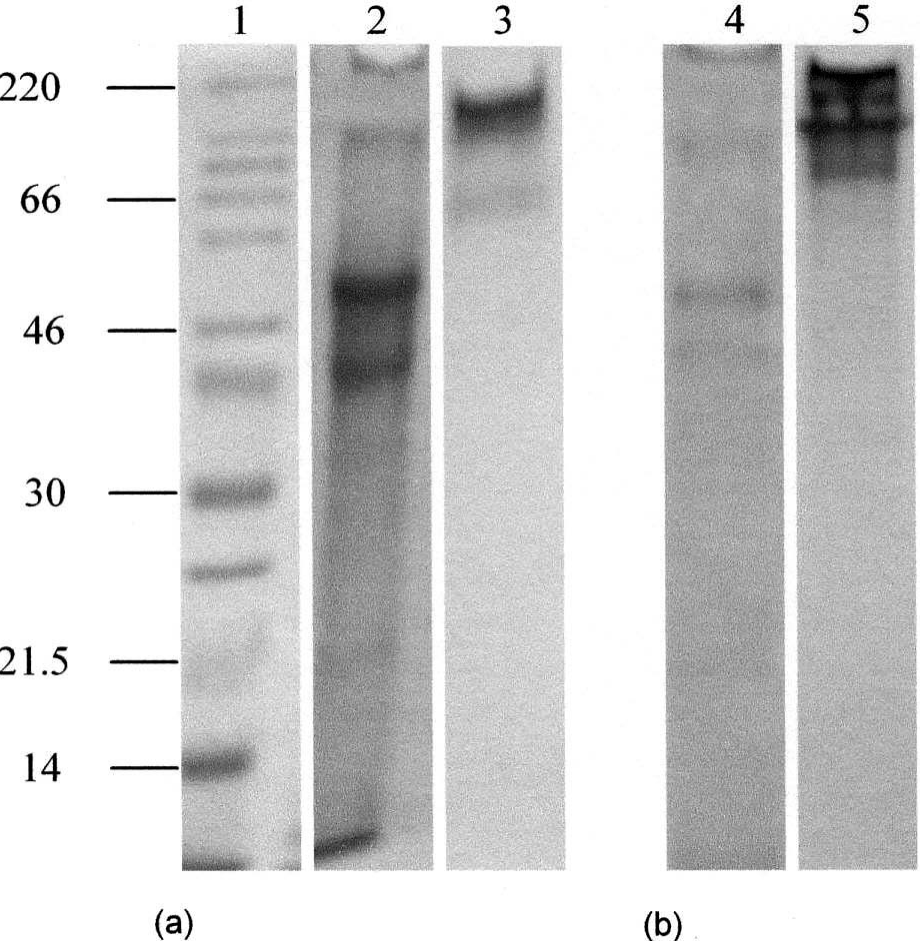
**SDS-PAGE analyses of the pregnancy plug mucins.** Freeze-dried pregnancy plug mucins (20 μg) before (a) and after (b) caesium chloride density gradient purification were separated on 10% SDS-PAGE and stained with Coomassie Brilliant Blue (lanes 1, 2 and 4) and PAS (lanes 3 and 5). Lane 1 is molecular weight marker in kDa.

Pregnancy plug mucus (20 μg) was dissolved in gel loading buffer containing 0.2 M 2-mercaptoethanol and loaded onto 10% SDS-PAGE (Fig. 2). Gels were stained either with PAS for carbohydrate or Coomassie Brilliant Blue G-250 for protein. An intense PAS positive band (*M*_*r *_>220 kDa) appeared on the top of the running gel below which there was another band of size <220 kDa (Fig. 2a, lane 3). Coomassie Blue staining also showed material at the top of the running gel and a number of bands of higher electrophoretic mobility and therefore of relatively smaller size within the gel (Fig. 2a, lane 2).

Caesium chloride density gradient ultra-centrifugation removed most of the contaminant protein from crude mucus as shown clearly by subsequent gel electrophoresis (Fig. 2b, lane 4). Bands at the top of the running gel, staining both for protein and carbohydrate confirmed the presence of the mucin and its purity (Fig. 2b, lanes 4 and 5).

### Western blotting

**Figure 3:**
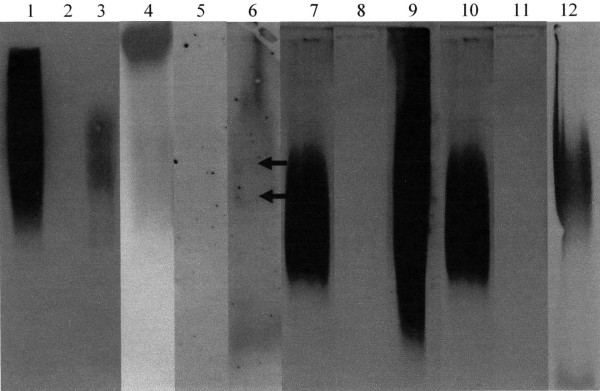
**Western blotting analyses of the purified pregnancy plug mucins.** Lane 1, MUC1 (positive control), lane 2, salivary MUC5B (negative control), lane 4, colonic mucus (positive control), lane 5, tracheal sputum (negative control), lane 7, pseudomyxoma peritonei (positive control), lane 8, salivary MUC7 (negative control), lane 10, pseudomyxoma peritonei (positive control), lane 11, gastric mucus (negative control) and lanes 3, 6, 9 and 12 purified pregnancy plug mucins were separated by a 1% agarose gel and transferred to nitrocellulose membrane. Following overnight blocking, the membranes were incubated for 2 h with mouse anti-MUC1 monoclonal (lanes 1, 2 and 3) and rabbit anti-MUC2 (lanes 4, 5 and 6), rabbit anti-MUC5AC (lanes 7, 8 and 9) and rabbit anti-MUC5B (lanes 10, 11 and 12) polyclonal antibodies. Membranes were then incubated for 1 h with HRPO linked goat anti-mouse and goat anti-rabbit secondary antibodies and bands that interacted with the antibodies were detected by ECL detection. NB the two bands of MUC2 (lane 6) are indicated by the arrows.

Western blot analysis was performed to determine the identity of the mucins present in the pregnancy plug mucus. Samples (40 μg each) were loaded on a 1% agarose gel and subjected to electrophoresis. Mucins were then transferred from the gel to a nitrocellulose membrane and probed with mouse anti-MUC1 monoclonal (Fig. 3 lanes 1, 2 and 3) and rabbit anti-MUC2 (lanes 4, 5 and 6), rabbit anti-MUC5AC (lanes 7, 8 and 9) and rabbit anti-MUC5B (lanes 10, 11 and 12) polyclonal antibodies. The Western blotting result confirmed the presence of MUC1, MUC2, MUC5AC and MUC5B mucins in the pregnancy plug mucus (Fig. 3 lanes 3, 6, 9 and 12 respectively). While MUC5AC was strongly expressed (Fig. 3 lane 9) MUC2 appeared in relatively smaller amounts and as a doublet (Fig. 3 lane 6, arrows) [19]. While the positive controls MUC1 (lane 1), colonic mucus (lane 4), pseudomyxoma peritonei (lanes 7 and 10) [20] reacted with the anti-MUC1, anti-MUC2, anti-MUC5AC and anti-MUC5B antibodies respectively, the negative controls namely the salivary MUC5B (lane 2), tracheal sputum (lane 5), salivary MUC7 (lane 8) and gastric mucus (lane 11) did not react with the anti-MUC1, anti-MUC2, anti-MUC5AC and anti-MUC5B antibodies respectively.

However, due to the lack of Western blotting antibodies against MUC4 and MUC6 the identification of these mucins was not done in this study.

### Toxicity assay

Prior to the HIV inhibition assay the toxicity of the crude pregnancy plug mucus and purified pregnancy plug mucins to the CEM SS cells was determined by toxicity assay. As shown in Table 1, no toxicity of these components or no cell death was detected.

**Table 1:** Toxicity of crude pregnancy plug mucus and purified pregnancy plug mucins to CEM SS cells.

Sample	Con	CEM SS cells	% of dead cells	% of live cells
Pregnancy plug mucus	0.9 mg	2.5 × 10^6^/ml	0	100
Pregnancy plug mucins	0.9 mg	2.5 × 10^6^/ml	0	100

### Inhibition assay

**Figure 4:**
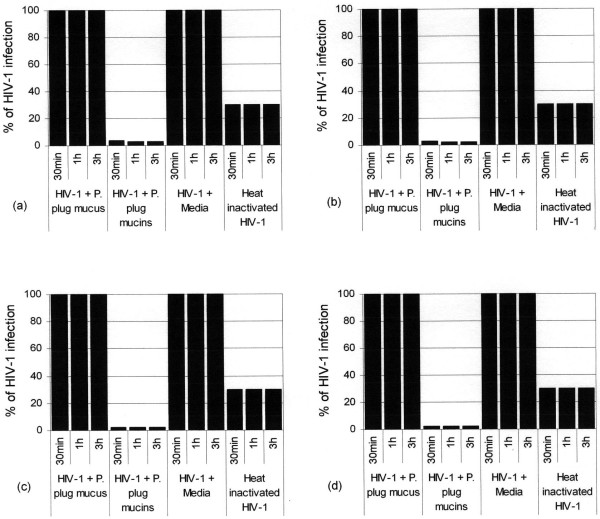
**Inhibition of HIV-1 activity by crude pregnancy plug mucus and purified pregnancy plug mucins *in vitro *assay.** Crude pregnancy plug mucus and purified pregnancy plug mucins (0.9 mg each) were incubated with subtype D HIV-1 for 60 min and filtered through 0.45 μm pore size cellulose acetate filter. As controls HIV-1 treated with media and heat inactivated HIV-1 were used. The unfiltered samples were then incubated with CEM SS cells at a concentration of 0.5 × 10^6^cells ml^-1 ^for 30 min, 1 h and 3 h. After PBS wash cells were cultured and viral replication was measured by a qualitative p24 antigen assay. Letters a, b, c and d indicate the anti-HIV-1 activity of each sample in a serial tenfold dilution of 10^-1^, 10^-2^, 10^-3 ^and 10^-4 ^respectively. P. plug represents pregnancy plug.

The anti-HIV-1 activities of the crude pregnancy plug mucus and purified pregnancy plug mucins were determined by HIV inhibition assay. When HIV-1 was incubated with crude pregnancy plug mucus for an hour and the mixture subsequently added to or incubated with the CEM SS cells for 30 min, a 100% HIV-1 infection of the CEM SS cells was measured by the p24 antigen assay (Fig. 4). However, when the virus was first incubated with purified mucins from the pregnancy plug for an hour and then the mixture subsequently incubated with the CEM SS cells for 30 min, an approximately 97.5% inhibition of the viral activity or an approximately 2.5% infection of the CEM SS cells was detected. This suggests that compared to the crude pregnancy plug mucus the purified pregnancy plug mucins reduce the infection of CEM SS cells by an approximately 39 fold (Fig. 4).

To determine the effect of time (incubation period) on the rate of viral infection or inhibition ability of the samples, the mixtures of (HIV-1 plus crude pregnancy plug mucus) and (HIV-1 plus purified pregnancy plug mucins) were incubated with the CEM SS cells for longer time periods (1 h and 3 h). However, no difference in the rate of viral infection or inhibition ability of the samples due to incubation time difference was observed (Fig. 4). To determine the anti-HIV-1 activity of the purified pregnancy plug mucins at the highest dilution or lowest concentration, serial tenfold fold dilutions (i.e. 10^-1^, 10^-2^, 10^-3 ^and 10^-4^) of the mucins were also done. Again, no difference in the anti-HIV-1 activity of the purified pregnancy plug mucins was detected down to10^-4 ^(Fig. 4a,b,c and 4d).

As shown in Fig. 4, when HIV-1 was incubated with the media (positive control) instead of the pregnancy plug mucins prior to addition to the CEM SS cells at all time points (30 min, 1 h and 3 h), HIV-1 infection of the CEM SS cells was not inhibited and 100% HIV-1 replication or infection of the CEM SS cells was measured by the p24 antigen assay. Surprisingly the heat inactivated HIV-1 (negative control) was also shown to cause an approximately 30% infection of the CEM SS cells at all time points (Fig. 4).

To determine or compare the efficiency of HIV-1 aggregation by the crude pregnancy plug mucus and purified pregnancy plug mucins, at the end of the incubation period (1 h), the mixtures of (HIV-1 plus crude pregnancy plug mucus), (HIV-1 plus purified pregnancy plug mucins) and the control (HIV-1 plus media) were filtered through 0.45 μm pore size cellulose acetate filter (25 mm diameter) and the filtrates were added to or incubated with the CEM SS cells at different time-points (30 min, 1 h and 3 h). The result demonstrated that the filtrates from the mixtures of (HIV-1 plus crude pregnancy plug mucus) and (HIV-1 plus media) caused 100% HIV-1 infection of the CEM SS cells (results not shown).

## Discussion

According to various studies [9,10,21,22], salivary macromolecules (possibly mucins) aggregate HIV-1 prior to host cell entry, thus preventing transmission of HIV-1 through saliva. Wiggins *et al*. [7] reported that mucus is the first line of defence against pathogenic micro-organisms. Studies in our laboratory have also confirmed these findings [11]. Crude saliva (from individuals with a self-declared risk free lifestyle and thus presumably uninfected), and its purified mucins MUC5B and MUC7 [11] and purified MUC1 from breast milk [12] show anti-HIV-1 activity in an *in vitro *inhibition assay.

It thus remains to be asked why other areas such as the female reproductive tract and breast milk, so rich in mucus and mucins quite similar in substance and conformation to those in saliva, still remain major routes of transmission of the virus. In the case of breast milk we showed that its MUC1 component inhibited the HIV-1 from infecting CEM SS cells in an *in vitro *assay only after it was dissociated from the milk fat globules and isolated and purified by caesium chloride density gradient ultra-centrifugation. Crude breast milk had no such inhibitory effect on HIV-1 [12]. In the light of this we decided to investigate whether cervical mucus and mucin display any anti-HIV-1 properties, considering that the cervix is a significant route of transmission in women.

The quality and quantity of cervical mucins during the different phases of the menstrual cycle are reported to vary either through the influence of oestrogen (proliferative phase) or of progesterone (luteal phase). For example the production of MUC5B was reported to increase at the mid-cycle and decrease during the secretory phase of the menstrual cycle whilst MUC4 increases during the luteal phase of the menstrual cycle [8,23]. These cyclical variations together with the fact that cervical scrapings, which yielded very small amounts of crude material made it difficult to investigate the anti-HIV-1 activity of these mucins per se. Therefore mucus plugs at the mouth of the cervix rich in mucin [2,14], were obtained from women in labour. However, a comparison of the effect of purified plug mucin versus purified cervical mucin on HIV is being planned.

In this study we have demonstrated that the purified mucins from the pregnancy plug inhibited HIV-1 infection of the CEM SS cells. However, the crude pregnancy plug mucus and the media failed to inhibit HIV-1 infection of these cells. Though the mechanism of inhibition is not clear, it is likely that when the HIV-1 was incubated with the mucins, the virus was trapped by aggregation through the sugar side-chains of the mucins, a purely physical phenomenon [10,24-26], resulting in preventing the virus from entering the host cells (CEM SS cells). This was supported by our finding that salivary MUC7 inhibited HIV-1 infection of the CEM SS cells when it was incubated with the virus prior to addition to the CEM SS cells. However, the mucin failed to inhibit viral infection of these cells when it was incubated with CEM SS cells prior to addition of the virus (unpublished data). This suggests that the mucin inhibits HIV-1 infection by physically aggregating the virus than by blocking putative viral binding sites or receptors on the cells.

The virus and mucins were incubated together with the cells for different incubation periods, i.e. 30 min, 1 h and 3 h to determine the effect of time on infection or lack thereof. Cultures were then washed three times after each incubation period to remove free virus and cultured for another 4 days in IL-2 rich media. This was done to determine if the virus had entered the cells during the initial incubation step and was able to replicate inside the cells for the extended incubation period to produce p24 antigen, or if the mucins were successful in preventing viral entry into the cells and therefore prevent the production of p24 antigen.

To further confirm the hypothesis that mucins inhibit HIV-1 activity by physically aggregating the virus, the CEM SS cells were incubated with the filtrates from the mixtures. The lower infection (2.5%) of the CEM-SS cells by the filtrate from the mixture of HIV-1 plus purified pregnancy plug mucins suggests the presence of insignificant amount of viruses in the filtrate or almost complete aggregation of the virus by the mucins, leaving no free viruses to pass through the filter paper into the filtrate to cause viral infection. On the other hand the 100% infection of the CEM-SS cells caused by the filtrates from the mixtures of HIV-1 plus crude pregnancy plug mucus and HIV-1 plus media suggests the presence of higher amount of viruses in the filtrates or the failure of the crude pregnancy plug mucus and the media to aggregate the viruses. This finding agreed with the report that HIV-1 may bind to the high-molecular weight components which results in macromolecular complex formation which is removable by filtration through 0.45 μm pore filter paper [10,24-26].

The lack of inhibition by crude pregnancy plug mucus compared to the inhibition by purified pregnancy plug mucins is not clear. However it should be considered that mucins constitute only about 0.5–1% of total crude mucus [27] which is known to contain water, glycoproteins, lipids, nucleic acids, lactoferrin, lysozyme, immunoglobulins and ions [7]. It is likely therefore that the potency of mucins would in this case be in their purified form rather than when they are a minor part of a larger secretion in which their concentration would be diluted. This was quite different in the case of crude saliva, the inhibitory effect of which was similar to that of its purified mucins, separable by gel filtration and individually effective against the virus [11]. However, quantification of the amount of mucins in the crude mucus prior to any assay should be considered before drawing this conclusion.

The heat inactivated HIV-1 (negative control) caused an approximately 30% infection of the CEM SS cells suggesting that the viruses, when inactivated but not completely killed are still infective, albeit to a lesser degree. To determine whether there is a dose/effect relationship and the lowest possible effective concentration with anti-HIV-1 activity, ten fold serial dilutions (10^-1^to 10^-4^) of the mucins were also done from a starting concentration of purified mucin of 0.9 mg. The mucins showed strong anti-HIV-1 activity down to a dilution of 10^-4^, but in this study the lowest possible concentration which can cause inhibition of HIV-1 activity was not identified. Thus a lower starting concentration of purified mucin than 0.9 mg would be advisable.

There was also no effect of time (incubation period) on the inhibitory effect of mucins or the infectivity of the virus. This suggested that the mucins aggregated the virus immediately and permanently. However, shorter starting times of incubation of mucins and the virus would be necessary to determine the shortest time mucins take to aggregate the virus.

Although HIV-1 Subtype C is currently the most prevalent in South Africa, the Subtype D which was used in this study was found during the early HIV epidemic in the country and is quite prevalent here, albeit to a lesser degree. Even though we wished to use the Subtype C strain, the Subtype D strain is unfortunately the only lab adapted strain we had available to us in the vicinity of Cape Town and it is possible that this is the only laboratory based HIV assay in the country. As described in the Methods section, this virus was first isolated from an AIDS patient by the Department of Medical Virology, Tygerberg Hospital, University of Stellenbosch, South Africa, in February 1988, and it was fully characterised and sequenced subsequently [28]. The human T lymphoblastoid cell line (CEM SS cells), which was used in this study, is reported to express CD4, CXCR4, ICAM-3 and MHC class II molecules [29]. These cells are capable of developing easily quantifiable syncytia formation in four to six days upon the addition of HIV-1 [30]. Although Subtype C predominantly uses CCR5, several instances of co-receptor switch to CXCR4 or even dual tropism have been observed in Subtype C, especially later in infection. Therefore this study could be relevant to *in vivo *situations, where transmitted viruses are most often CCR5 tropic.

Extraction of mucus was in 6 M GuHCl and proteolytic inhibitors which included 10 mM EDTA, 5 mM NEM, and 1 mM PMSF to reduce endogenous proteolysis of mucins [2]. PMSF and EDTA inhibit serine and metallo-protease activity respectively whilst NEM inhibits thiol proteases and minimizes thiol-disulfide exchange [1].

Caesium chloride density gradient purification removes all contaminants such as non-mucin proteins, lipids, proteoglycans and nucleic acids from mucins [31]. Purification of the mucins was confirmed by SDS-PAGE [32]. The removal of these contaminants from mucins was believed to be by dissociative conditions through the presence of GuHCl [1], known to be a widely used denaturant [33] which in this case could well dismantle the tertiary structure of mucins [14].

The presence of MUC1, MUC2, MUC5AC and MUC5B in the pregnancy plug mucus was confirmed by Western blotting with MUC2 expressed as a doublet and in small amount compared to the other mucins. Immunohistochemistry confirmed previous reports of the expression of MUC4 and MUC6 by the endometrial tissue (data not shown), but their presence in the mucus plug could not be confirmed due to the lack of antibodies to these mucins for Western blotting. This result agreed with that of Gipson *et al*. [6], Wiggins *et al*. [7], Gipson *et al*. [23] and Wickstrom *et al*. [34], studies which reported the expression of MUC1, MUC2, MUC4, MUC5AC, MUC5B and MUC6 by the female reproductive tract.

## Conclusion

In summary, we have shown the *in vitro *inhibition of HIV-1 activity by purified mucins from the pregnancy plug. However, the crude pregnancy plug mucus failed to inhibit HIV-1 activity. Although it is not clear why the crude sample did not inhibit HIV-1 activity, it is likely that the amount of mucins in the crude pregnancy plug mucus is of too low a concentration to cause viral inhibition or aggregation. Future studies will attempt to establish the lowest amount of purified mucin required to cause aggregation of the virus. Also different HIV strains, cell lines and samples from different donors for statistical validity to strengthen this preliminary finding, will be carried out. A comparison between the anti-HIV-1 activity of each cervical mucin from the different stages of the menstrual cycle has also been planned.

## Materials and methods

### Ethics

The University of Cape Town Research and Ethics Committee approved this study; ethics number REC REF: 283/2004

### Materials

Mouse anti-MUC1 monoclonal (NCL-MUC1, 201607) and goat anti-mouse horse radish peroxidise (HRPO) linked secondary antibodies (sc-2005) were from Novocastra (Newcastle, UK) and Santa Cruz (California, USA) respectively. Polyclonal rabbit anti-MUC2 (LUM2-3), anti-MUC5AC (LUM5-1), anti-MUC5B (LUM5B-2) and goat anti-rabbit HRPO linked secondary antibodies were kindly provided by Sara Kirkham (Manchester, UK). The CEM SS cells were from AIDS Research and Reference Reagent Programme (Germantown, USA). The p24 antigen kit was from Vironostika HIV-1 Antigen kit Biomérieux (France). Sepharose CL-4B and reagent solvents such as guanidinium chloride (GuHCl) and caesium chloride (CsCl) were from Sigma (UK). Trypan Blue Dye solution was from Merck (Germany).

### Pregnancy plug mucus collection

Pregnancy plug mucus was obtained from the Groote Schuur Hospital Maternity Division at the University of Cape Town. The pregnancy plug mucus was retrieved prior to delivery and collected into cold 6 M GuHCl containing proteolytic inhibitors, namely 10 mM EDTA, 5 mM NEM and 1 mM PMSF pH 6.5 and stored at -20°C.

### Mucus preparation

Crude pregnancy plug mucus was prepared according to the method of Carlstedt *et al*. [2]. The pregnancy plug mucus was collected into 0.1 M Tris-HCl, 2% (w/v) EDTA and 5 mM PMSF pH 7.5 and prepared for the HIV inhibition assay. After gentle stirring for 15 h at 4°C, insoluble materials were removed by high-speed centrifugation at 9 000 g for 2 h at 4°C. The supernatant was dialysed against three changes of distilled water at 4°C and freeze-dried.

### Mucin preparation

Pregnancy plug mucus was thawed and stirred gently for 15 h at 4°C in 6 M GuHCl and a cocktail of proteolytic inhibitors as described above. Insoluble materials were removed by high-speed centrifugation at 9 000 g for 2 h at 4°C. The soluble material was then pooled and subjected to density gradient ultra-centrifugation, twice for 48 h at a 105 000 g at 4°C in a Beckman L45 ultra-centrifuge [31]. Briefly, samples in 4 M GuHCl containing 10 mM EDTA, 5 mM NEM and 0.05% CHAPS pH 6.5 were adjusted to a density of 1.39 to 1.40 g/ml with caesium chloride prior to centrifugation. Mucin rich fractions were pooled, dialysed against three changes of distilled water at 4°C and freeze-dried.

### SDS-PAGE analysis

Pregnancy plug mucins (20 μg) were prepared in reducing gel loading buffer containing 2% sodium dodecyl sulfate (SDS), 10% glycerol, 0.01% bromophenol blue and 5% mercaptoethanol and boiled for 2 min prior to loading. Electrophoresis was performed by the method of Laemmli [35] in a 10% (w/v) running gel and a 4% (w/v) stacking gel using the Hoeffer Mighty Small mini-electrophoresis system. After electrophoresis gels were stained for carbohydrate with Periodic Acid Schiff (PAS) and for protein with Coomassie Brilliant Blue G-250.

### Agarose gel electrophoresis

Purified pregnancy plug mucins (40 μg) were prepared in a sample loading buffer containing 40% glycerol, 0.01% bromophenol blue and 5% mercaptoethanol in 1 × Tris-acetate buffer (TAE) and boiled for 2 min prior to loading. Electrophoresis was carried out according to the method of Thornton *et al*. [36], in a 1% (w/v) agarose gel (15 × 15 cm) prepared in running buffer containing 40 mM TAE, 1 mM EDTA, and 0.1% SDS pH 8.0. Briefly, agarose (1.6 g in 160 ml of running buffer) was boiled in a microwave until completely dissolved and cooled down to approximately 50°C before pouring into the Bio-Rad DNA sub cell gel apparatus. Upon polymerization the apparatus was filled with running buffer and electrophoresis was performed at 100 V for 2.5 h at room temperature.

### Western blotting

After agarose gel electrophoresis the purified pregnancy plug mucins were transferred to nitrocellulose membrane (Nitrocellulose, 0.22 μ) by vacuum blotting for 1 h at a suction pressure of 40 mbar, according to the method of Thornton *et al*. [36]. The transfer buffer contained 4 × SSC (0.6 M NaCl, 60 mM Tri-sodium citrate, pH 7.0). After electro-blotting non-specific binding was blocked by incubating the membranes overnight in 5% (m/v) low fat milk powder in TBS, 0.05% Tween-20 (TBST) at 4°C. The membranes were then washed with TBST 3 × 5 min and incubated for 2 h with mouse anti-MUC1 monoclonal and rabbit anti-MUC2, anti-MUC5AC and anti-MUC5B polyclonal antibodies diluted in 5% (m/v) low fat milk powder in TBST at a dilution of 1 in 100 (mouse anti-MUC1), 1 in 5000 (rabbit anti-MUC2 and anti-MUC5AC) and 1 in 2000 (rabbit anti-MUC5B). The membranes were washed 3 × 5 min with TBST and incubated for 1 h with HRPO linked goat anti-mouse and goat anti-rabbit secondary antibodies diluted in 5% (m/v) low fat milk powder in TBST at 1 in 1500 and 1 in 2000 respectively. After another TBST wash (3 × 5 min) bands that interacted with the antibody were detected by exposing the membranes to ECL detection kit.

### Toxicity assay

The toxicity of crude pregnancy plug mucus and purified pregnancy plug mucins to the phytohaemagglutinin (PHA) stimulated CEM SS cells was tested. Briefly 500 μl of the CEM SS cells in RPMI complete containing 10% Fetal Calf Serum, 1% Penicillin/Streptomycin antibiotic, 10 μmol Fungin and 50 μmol 2-mercaptoethanol (final concentration 2.5 × 10^6 ^cells/ml) were incubated with 250 μl of IL-2 and 250 μl (0.9 mg) of crude pregnancy plug mucus and purified pregnancy plug mucins in CO_2 _incubator for 24 h. As controls CEM SS cells with IL-2 only and IL-2 without CEM SS cells (blank) were used. After spinning at 100 g for 5 min cells were re-suspended in 500 μl of RPMI and live and dead cells were counted using Trypan blue exclusion criteria. The percentage of viable cells was calculated as live cells/total cells × 100.

### HIV inhibition assay

The anti-HIV-1 activities of the crude pregnancy plug mucus and purified pregnancy plug mucins from HIV negative pregnant women were tested in an inhibition assay according to the method of Nagashunmugam *et al*. [10]. Briefly the crude pregnancy plug mucus and purified pregnancy plug mucins were dissolved in 0.25% PBS and (500 μl or 0.9 mg each) were mixed with 4 ml of the subtype D HIV-1 supernatant fluid (SNF) and incubated for 60 min at 37°C separately. As controls heat inactivated HIV-1 and HIV-1 plus media (RPMI 1640 with 10% fetal calf serum and IL-2) were used. The virus was first isolated from an AIDS patient by the Department of Medical Virology, Tygerberg Hospital, in February 1988, and it was fully characterised and sequenced subsequently [28]. At the end of the incubation period the mixtures (HIV-1 plus crude pregnancy plug mucus), (HIV-1 plus purified pregnancy plug mucins) and the control (HIV-1 plus media) were filtered through 0.45 μm pore size cellulose acetate filter (25 mm diameter) and both the unfiltered and filtered samples were incubated with the CEM SS cells at 37°C at a concentration of 0.5 × 10^6 ^cells/ml for 30 min, 1 h and 3 h. Cells were then washed three times with PBS to remove free virus and cultured. Supernatant fluid was harvested on Day 4 and viral replication was measured by a qualitative p24 antigen assay. Endpoints were calculated by the Reed-Muench formula and the 50% tissue culture infective dose (TCID_50_) was expressed as the highest dilution that produced a positive qualitative p24 antigen result. All samples were done in triplicate and the anti-HIV-1 activity of mucins was tested in a serial tenfold dilution (10^-1 ^to 10^-4^).

### Analytical determinations

Glycoprotein was estimated by the PAS procedure of Mantle and Allen [37] and protein according to the method of Lowry *et al*. [38].

## Competing interests

The authors declare that have no competing interests

## Authors' contributions

HHH carried out the biochemical studies and drafted the manuscript. CdB established and carried out the HIV inhibition assay. ZEL and MGT participated in the biochemical studies. LS participated in pregnancy plug mucus collection and analysis. DK contributed ideas to the design and coordination of the study. ASM conceived of the study, participated in its design and coordination and finalised the manuscript. All authors read and approved the final manuscript.
